# Entangled radicals may explain lithium effects on hyperactivity

**DOI:** 10.1038/s41598-021-91388-9

**Published:** 2021-06-09

**Authors:** Hadi Zadeh-Haghighi, Christoph Simon

**Affiliations:** 1 Department of Physics and Astronomy, University of Calgary, Calgary, AB T2N 1N4 Canada; 2 Institute for Quantum Science and Technology, University of Calgary, Calgary, AB T2N 1N4 Canada; 3 Quantum Alberta, University of Calgary, Calgary, AB T2N 1N4 Canada; 4 Hotchkiss Brain Institute, University of Calgary, Calgary, AB T2N 1N4 Canada

**Keywords:** Computational biophysics, Neuroscience, Molecular medicine, Biological physics, Quantum physics

## Abstract

It is known that bipolar disorder and its lithium treatment involve the modulation of oxidative stress. Moreover, it has been observed that lithium’s effects are isotope-dependent. Based on these findings, here we propose that lithium exerts its effects by influencing the recombination dynamics of a naturally occurring radical pair involving oxygen. We develop a simple model inspired by the radical-pair mechanism in cryptochrome in the context of avian magnetoreception and xenon-induced anesthesia. Our model reproduces the observed isotopic dependence in the lithium treatment of hyperactivity in rats. It predicts a magnetic-field dependence of the effectiveness of lithium, which provides one potential experimental test of our hypothesis. Our findings show that Nature might harness quantum entanglement for the brain’s cognitive processes.

## Introduction

The human brain is a magnificent system with highly complex functionalities such as learning, memory, emotion, and subjective experience, each of which is *mood* dependent^1^. Everyday millions of patients all over the world take psychiatric pharmaceuticals to stabilize their mood, yet the underlying mechanisms behind these medications remain largely unknown^2^.

Bipolar disorder (BD) is a devastating mental illness which affects 2–4% of the world population^3^. As its name implies, it entails two distinct oscillating and opposing states, a state of energy and hyperactivity (the manic phase), and a state of low energy (depression)^4^. Lithium (Li) administration is the first-line treatment of bipolar illness^3,5–7^. Despite the common clinical use of this drug, the mechanism by which it exerts its effects remains elusive^8,9^. Here, based on experimental findings, we propose a mechanism for Li’s therapeutic effects on BD.

Studies in the 1980s showed that administering Li results in different parenting behaviors and potentially delayed offspring development in rats^10^. Although these findings weren’t quantitative, it was reported that different Li isotopes have different impacts. Recently, a new study was conducted by Ettenberg et al.^11^ demonstrating an isotope effect of Li on the manic phase in rats. Li has two stable isotopes, ${^{6}}$Li and ${^{7}}$Li, which carry different nuclear spin angular momentum, $I_6=1$ and $I_7=3/2$, respectively. In that study, sub-anesthetic doses of ketamine were administered to induce hyperactivity which was then treated with lithium. The findings of that work indicate that ${^{6}}$Li produces a longer suppression of hyperactivity in an animal model of mania compared to ${^{7}}$Li.

Moreover, there is accumulating evidence indicating that BD^12–19^ and its Li treatment^7,15,20,21^ are associated with oxidative stress, which is an imbalance between production and accumulation of radical oxygen species (ROS) in cells and tissues and the ability of a biological system to detoxify these reactive products, essential for governing life processes^22^. It therefore seems pertinent to explore the relationship between nuclear spin effects and oxidative stress in lithium mania therapy.

Any proposed mechanism for Li’s BD treatment should embrace these two facts: Li’s effect on BD exhibits isotopic dependence, and BD is connected to abnormalities in oxidative stress. In other words, the balance of naturally occurring free radicals is disrupted in BD patients, and lithium’s different isotopes appear to alter the free radical formations differently. It is therefore possible that nuclear spin properties might be the key for the differential effects of the two lithium isotopes in BD. Here, we propose that lithium affects naturally occurring radical pairs (RPs) in a way that is modulated by the hyperfine interaction (HFI).

Spins can play crucial roles in chemical reactions even though the energies involved are orders of magnitude smaller than the thermal energy, $k_BT$^23,24^. It has been known since the 1970s that external magnetic fields and nuclear spins can alter the rates and product yields of certain chemical reactions^25,26^. The key ingredients are RPs created simultaneously, such that the two electron spins, one on each radical, are entangled. Organic radicals are typically created in singlet (S) or triplet (T) entangled states by a reaction that conserves electron spin. Any spin in the vicinity or an external magnetic field can alter the extent and timing of the S-T interchange and hence the yields of products formed spin-selectively from the S and T states^27,28^.

Over the past decades, it has been proposed that that quantum physics could help answer unsolved questions in life science^29–31^. The above-described Radical Pair Mechanism (RPM) is one of the most well-established models in quantum biology; it could give a promising explanation for how migratory birds can navigate by means of Earth’s magnetic field^32,33^. The application of RPM has begun to gain momentum in numerous fields of research^34^. Most recently, Smith et al., inspired by the the RPM explanation of avian magnetoreception, have shown that the RPM could play a role in xenon-induced anesthesia, which exhibits isotopic dependence^35^.

In the context of avian magnetoreception, the RPM is thought to involve the protein cryptochrome (Cry)^36^, which contains the flavin adenine dinucleotide (FAD) cofactor. It is known that in Cry RPs can be in the form of flavosemiquinone radical (FAD${^{.-}}$) and terminal tryptophan radical (TrpH${^{.+}}$)^32,37,38^. However, considerable evidence suggests that the superoxide radical, O2${^{.-}}$, can be an alternative partner for the flavin radical, such that FADH${^{.}}$ and O2${^{.-}}$ act as the donor and the acceptor, respectively^39–42^. This is motivated by the fact that the magnetically sensitive step in the reaction scheme occurs in the dark^43^, with required prior exposure to light, and that a strongly asymmetric distribution of hyperfine couplings across the two radicals results in a stronger magnetic field effect than a more even distribution^44^. Furthermore, Zhang et al.^45^ recently showed that a magnetic field much weaker than that of the Earth attenuates adult hippocampal neurogenesis and cognition and that this effect is mediated by ROS. Additionally, it has been shown that the biological production of ROS *in vivo* can be influenced by oscillating magnetic fields at Zeeman resonance, indicating coherent S-T mixing in the ROS formation^46^.

BD is characterized by shifts in energy, activity, and mood and is correlated with disruptions in circadian rhythms^47,48^ and abnormalities in oxidative stress^12–19^. It is known that Li influences the circadian clock in humans, and circadian rhythms are disrupted in patients with BD for which Li is a common treatment^49,50^. Yet, the exact mechanisms and pathways behind this therapy are under debate. However, it has been shown that Li acts directly on the mammalian suprachiasmatic nucleus (SCN), a circadian pacemaker in the brain^51,52^. Cry’s are necessary for SCN’s development of intercellular networks that subserve coherent rhythm expression^53^. Further, it has also been reported that Cry2 is associated with BD^54^. In addition, Cry’s essential role has been discovered in axon outgrowth in low intensity repetitive transcranial magnetic stimulation (rTMS)^55^. It thus seems that Cry might also have vital roles in the brain’s functionalities related to BD. The [FADH${^{.}}$... O2${^{.-}}$] RP formation in Cry is consistent with evidence that Li treatment^7,15,20,21^ and BD^12–19^ are associated with oxidative stress implying a key role for radicals in BD and Li effects. Furthermore, it has also be shown that weak-pulsed electromagnetic fields have impacts on Cry of mammalian cells and cause increased ROS production, which appeared to depend on Cry^56^. In a subsequent study, it was demonstrated that the application of an extremely low-frequency magnetic field influences the cellular clocks^57^. Motivated by these results, in the present work, we specifically focus on the Cry pathway for Li therapy, however, this is not the only way in which radical pairs could play a role in lithium effects on BD. It has also been suggested that Li competes with magnesium (Mg) in inhibiting Glycogen synthase kinase-3^58^ and at binding sites of the N-methyl-D-aspartate (NMDA) receptor^59^, however, the existence of naturally occurring RPs in these target proteins requires experimental support. Alternative targets will be discussed briefly in the Discussion section.

**Figure 1:**
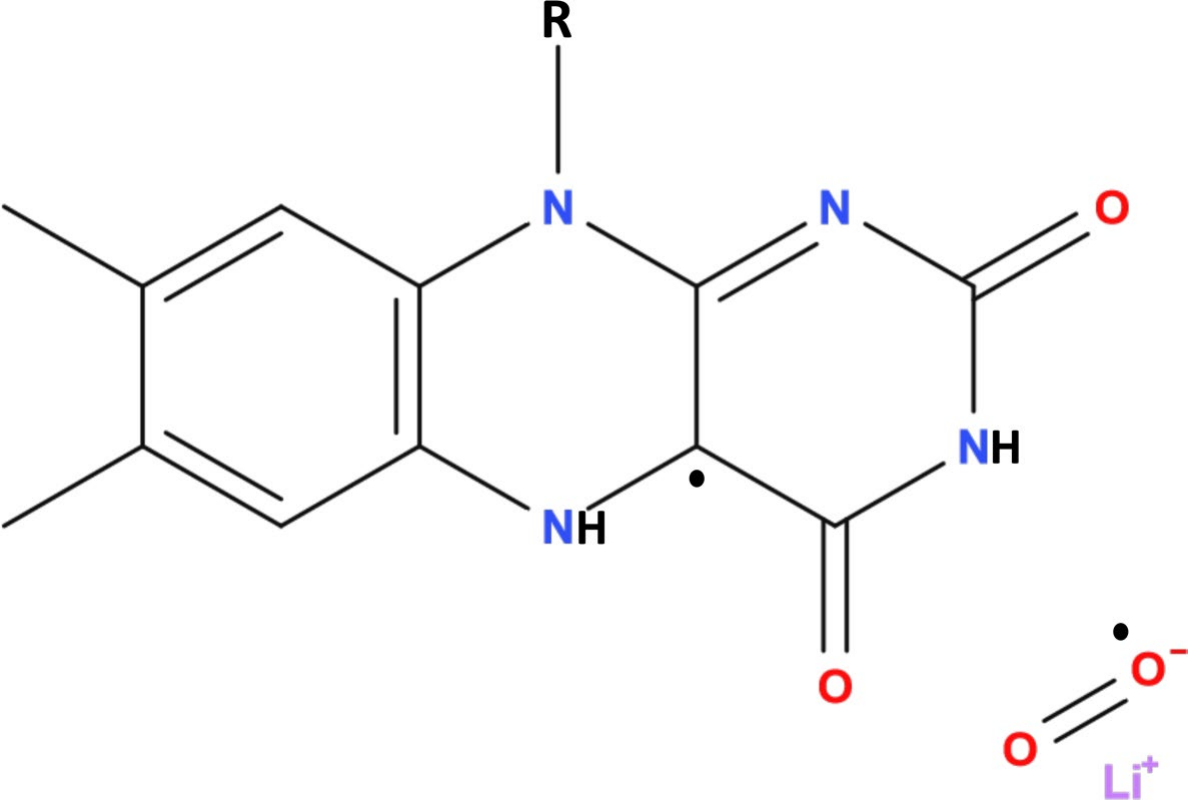
Flavinsemiquinone [FADH${^{.}}$] and lithium superoxide [Li${^{+}}$-O2${^{.-}}$] radical pair. The radical pair undergoes intersystem crossing between singlet and triplet entangled states.

Here, we propose that the RPM could be the underlying mechanism behind the isotope effects of Li treatment for hyperactivity. We propose that Li’s nuclear spin modulates the recombination dynamics of S-T interconversion in the naturally occurring RPs in the [FADH${^{.}}$... O2${^{.-}}$] complex, and due to the distinct nuclear spins, each isotope of Li influences these dynamics differently, which results in different therapeutic effects, see Fig. 1.

Let us note that an alternative interpretation has also been proposed for the isotope dependence in lithium’s effect on mania. Fisher has suggested that phosphorus nuclear spins could be entangled in networks of Posner molecules, ${Ca}_{9}({PO}_4)_6$, which could form the basis of a quantum mechanism for neural processing in the brain^60^. Replacement of calcium at the center of a Posner molecule with another cation can further stabilize the Posner molecule^61^, where each isotope of the cation, due to their different nuclear spins, has a different impact. This also provides a potential interpretation for the lithium isotope dependence treatment for mania. However, this model demands more supporting evidence and has been recently challenged experimentally^62^. Moreover, our proposed explanation based on the RPM allows us to make connections with the essential roles of ROS^45^ and Cry^55^ in the brain’s neuronal activity and in the biological pathways for lithium effects on BD. In the following, we aim to probe the possibility of RPM for lithium treatment and analyze the parameter values that are required in order to explain the isotopic effect of Li’s BD treatment observed by Ettenberg et al.^11^.

## Results

### Lithium effectiveness on hyperactivity and RPM

#### Quantifying effectiveness

Ettenberg et al.^11^ recorded the effects of lithium treatments on ketamine-induced locomotor activity of rats. They conducted the test over 60 min sessions beginning immediately after ketamine injection. After administering of ${^{6}}$Li and ${^{7}}$Li, they measured the mean traveled distance with the standard error of the mean (SEM) for every 5 min for a group of 16 rats for each isotope treatment. They observed that ${^{6}}$Li treatment exhibited significantly greater and more prolonged reductions in hyperactivity compared to ${^{7}}$Li. Here we define the cumulative traveled distance in 60 min $TD_6$ and $TD_7$, respectively, for ${^{6}}$Li and ${^{7}}$Li. The total traveled distance ratio $TD_r$ is defined such that $TD_r = TD_7/TD_6$, see Table 1. We derived the uncertainties based on the reported mean (±SEM) hyperlocomotion from Ettenberg et al.^11^ using standard error propagation.

**Table 1:** Li isotopic nuclear spins and total traveled distance in 60 min for ${^{6}}$Li and ${^{7}}$Li, $TD_6$ and $TD_7$, respectively, taken form the work of Ettenberg et al.^11^. $TD_r$ is the total traveled distance ratio.

Treatment	Total traveled distance (cm)	Uncertainty
${^{6}}$Li ($I=1$)	$TD_6=20112$	$\pm 4.62\%$
${^{7}}$Li ($I=3/2$)	$TD_7=25630$	$\pm 3.83\%$
	$$TD_r=TD_7/TD_6=1.3\pm 6\%$$	

#### RPM model

The RP model developed here is used to reproduce the effectiveness of Li for treatment of hyperactivity based on alteration in the triplet yield for different isotopes. Here, we propose that Li may interact with the RP system of FADH and superoxide. The correlated spins of RP are assumed to be in [FADH${^{.}}$... O2${^{.-}}$], where the unpaired electron on O2${^{.-}}$ couples with the Li nuclear spin, see Fig. 1.

In this model, we consider a simplified form of interactions for the RPM by only including Zeeman and HF interactions^32,63^. Given the likely randomized orientation of the relevant proteins, for the HFIs, we only consider the isotropic Fermi contact contributions. In our calculations, we assume that the unpaired electron on FADH${^{.}}$ couples only with the isoalloxazine nitrogen nucleus, which has the largest isotropic HF coupling constant (HFCC) among all the atoms in FAD^64^, following the work of Hore^65^, and that the other unpaired electron on [Li${^{+}}$-O2${^{.-}}$] couples with the lithium nucleus. The Hamiltonian for our RP system reads as follows:

1 $$\begin{aligned} {\hat{H}}=\omega \hat{S}_{A_{z}}+a_1 \hat{\mathbf {S}}_A.\hat{\mathbf {I}}_1+\omega \hat{S}_{B_{z}}+a_2 \hat{\mathbf {S}}_B.\hat{\mathbf {I}}_2, \end{aligned}$$

where $\hat{\mathbf {S}}_A$ and $\hat{\mathbf {S}}_B$ are the spin operators of radical electron A and B, respectively, $\hat{\mathbf {I}}_1$ is the nuclear spin operator of the isoalloxazine nitrogen of FADH${^{.}}$, $\hat{\mathbf {I}}_2$ is the nuclear spin operator of the Li nucleus, $a_1$ is the HFCC between the isoalloxazine nitrogen of FADH${^{.}}$ and the radical electron A ($a_1=523.3 \mu T$^64^), $a_2$ is the HFCC between the Li nucleus and the radical electron B, and $\omega$ is the Larmor precession frequency of the electrons due to the Zeeman effect. Of note, there is no need to include any potential competition with Mg explicitly in the model as we are interested in comparing the Li isotopes.

#### DFT

**Figure 2:**
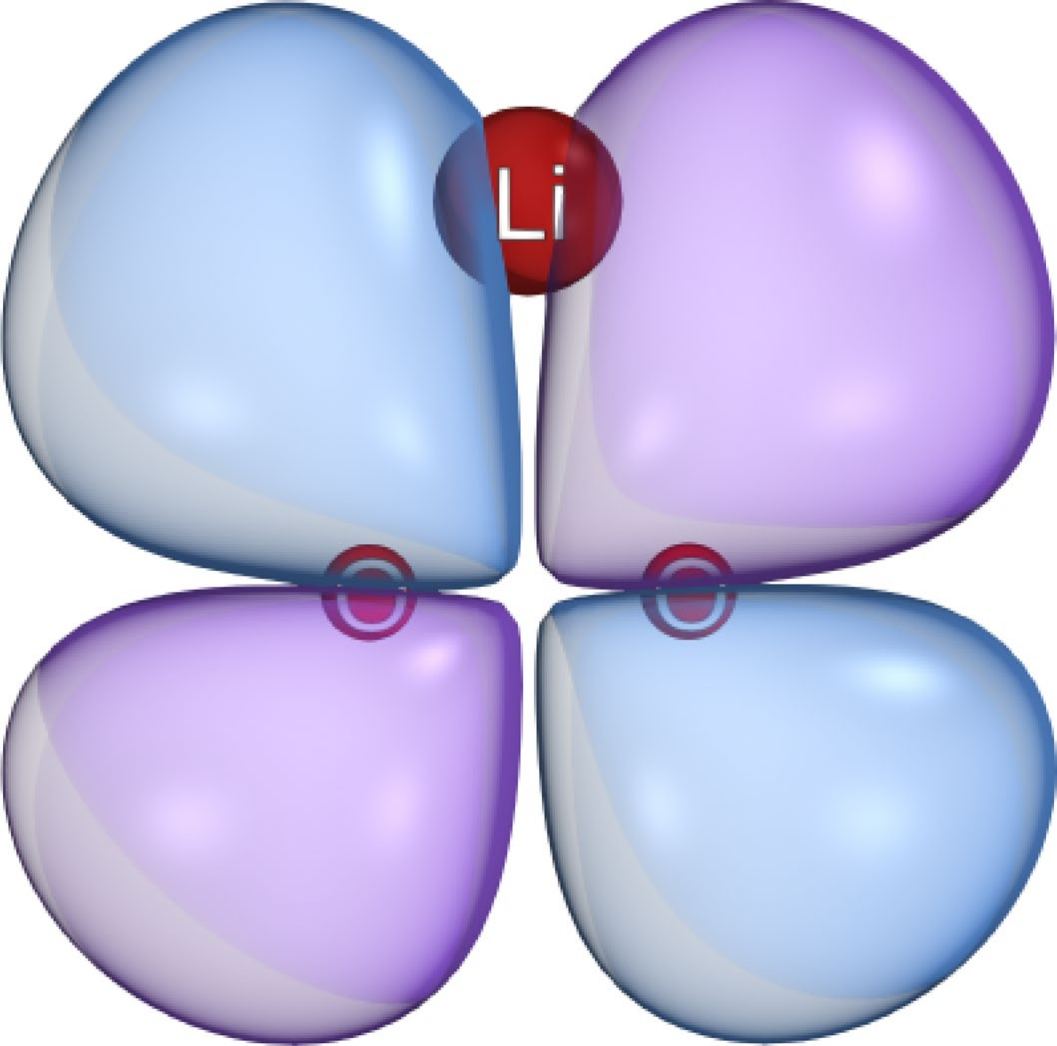
The highest occupied molecular orbital of [Li${^{+}}$-O2${^{.-}}$]. Imaged rendered using IboView v20150427 2 (http://www.iboview.org).

We use density functional theory (DFT) to determine the reasonable range for the Li HFCC. Our DFT calculations show that the unpaired electron of [Li${^{+}}$-O2${^{.-}}$] is bound. The the highest occupied molecular orbital (HOMO) is shown in Fig. 2. The resulting Mulliken charge and spin population of the [Li${^{+}}$-O2${^{.-}}$] complex indicates that the unpaired electron resides primarily on the O2 molecule but is extended slightly onto the lithium atom, see Table 2.

**Table 2:** Mulliken charge and spin population of [Li${^{+}}$... O2${^{.-}}$].

Atom	Charge population	Spin population
O	$-$0.244409	0.524734
O	$-$0.244398	0.524744
Li	0.488807	$-$0.049479
Sum	0	1

Using different DFT functionals and basis-sets, we find a range of values for the HFCC of Li nucleus at the distance of $\sim 1.6 \text {\AA }$ from O2${^{.-}}$, which is close to the values from other studies^66^, $|a_2| \in [157.7, 282] \mu$T. The HFCC values between the unpaired electron on FADH${^{.}}$ and the isoalloxazine nitrogen nuclear spin are taken from Maeda et al.^64^.

#### Triplet yield calculation

The triplet yield produced by the radical pair reaction can be obtained by tracking the spin state of the radical pair over the course of the reaction. This can be carried out by solving the Liouville-von Neumann equation, which describes the evolution of the density matrix over time. The eigenvalues and eigenvectors of the Hamiltonian can be used to determine the ultimate triplet yield ($\Phi _T$) for time periods much greater than the RP lifetime:

2 $$\begin{aligned} \Phi _T=\frac{3(k+r)+k}{4(k+r)}-\frac{1}{M}\sum _{m,n=1}^{4M} \frac{\left\| {\langle {m}|}\hat{P}^S {|{n}\rangle }\right\| ^2 k(k+r)}{(k+r)^2+(\omega _m-\omega _n)^2}, \end{aligned}$$

where *M* is the total number of nuclear spin configurations, $\hat{P}^S$ is the singlet projection operator, ${|{m}\rangle }$ and ${|{n}\rangle }$ are eigenstates of $\hat{H}$ with corresponding eigenenergies of $\omega _m$ and $\omega _n$, respectively, *k* is the RP lifetime rate, and *r* is the RP spin-coherence lifetime rate.

**Figure 3:**
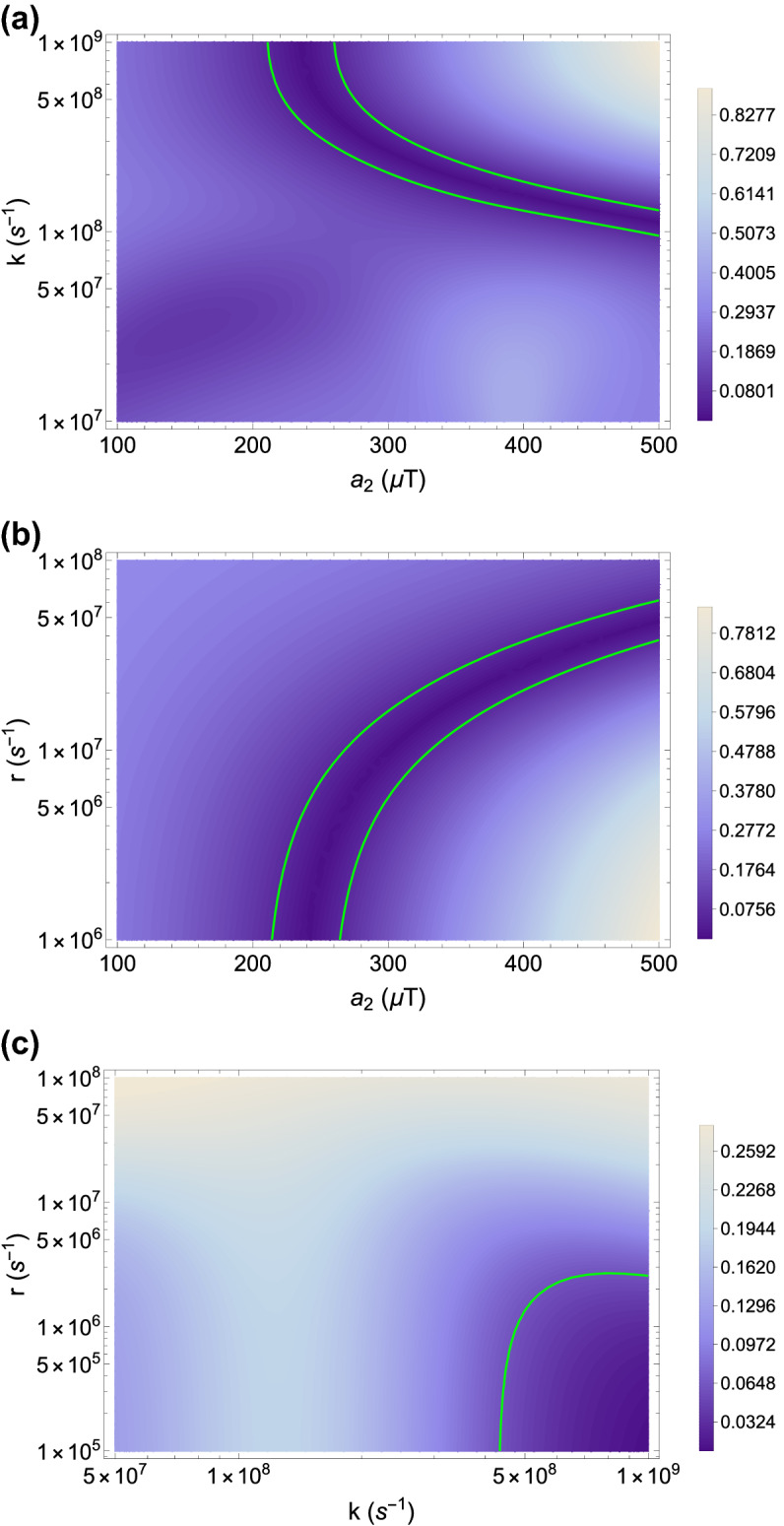
The dependence of the agreement between the total traveled distance ratio, $TD_r$, and the triplet yield ratio, $TY_r$ of ${^{7}}$Li over ${^{6}}$Li on the relationship between: (**a**) the radical pair reaction rate, *k*, and the lithium hyperfine coupling constant, $|a_2|$, for $r = 1.0\times 10^6$ s$^{-1}$ (**b**) the radical pair spin-coherence relaxation rate, *r*, and the lithium hyperfine coupling constant, $|a_2|$, for $k = 7.0\times 10^8$ s$^{-1}$ and (**c**) the RP reaction rate, *k* and the radical pair spin-coherence relaxation rate, *r*, for $|a_2| =224.4 \mu$T. In all three cases $a_1 =523.3 \mu$T and $B = 50 \mu$T. The absolute value of the difference between the prediction of radical pair mechanism, $TY_r$, and the experimental data, $TD_r$, is illustrated where the green line indicates the ranges smaller than the experimental uncertainty, $|TD_r-TY_r| \le 0.06$.

**Figure 4:**
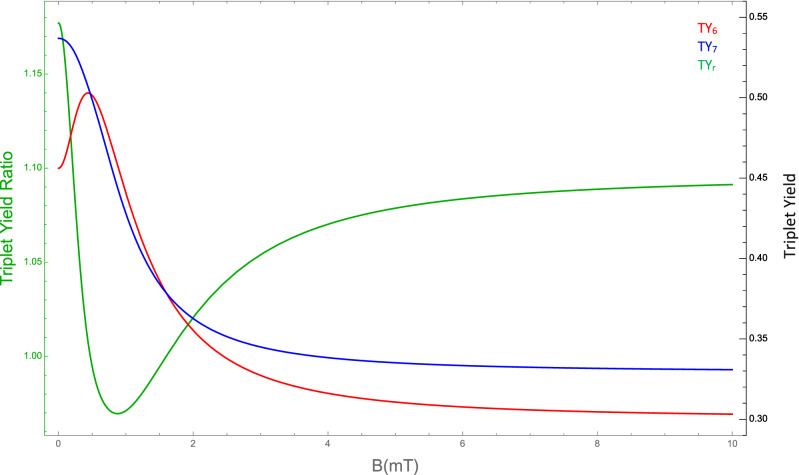
The dependence of the triplet yield of ${^{6}}$Li, $TY_6$, (red) and ${^{7}}$Li, $TY_7$, (blue) and the triplet yield ratio, $TY_r$, (green) on external magnetic field *B* for $a_1 = 523.3 \mu$T, $|a_2| = 224.4 \mu$T, $r = 1.0\times 10^6$ s$^{-1}$, and $k= 4.0\times 10^7$ s$^{-1}$.

Here we explore the sensitivity of the triplet yield ratio to changes in the HFCC $a_2$ between unpaired electron B and the lithium nucleus, external magnetic field strength (*B*), RP reaction rate (*k*), and RP spin-coherence relaxation rate (*r*). For the comparison between the experimental measurements and our RPM model, the absolute value of the difference between total traveled distance ratio and triplet yield, $|TD_r-TY_r|$, is presented on the $|a_2|$ and *k* plane in Fig. 3a, on the $|a_2|$ and *r* plane in Fig. 3b, and on the *k* and *r* plane in Fig. 3c. The dependence of the *TY* for each isotope of lithium and their ratio on the external magnetic field is shown in Fig. 4. The experimental findings of the isotopic-dependence of Li treatment for hyperactivity are reproducible for $B\in [0,200] \mu$T, which includes the geomagnetic field at different geographic locations (25 to $65 \mu$T)^67^. We aim to find regions in parameter space by which the triplet yield calculated from our RPM model fits with the experimental findings on the isotope dependence of the effectiveness of lithium for hyperactivity treatment. We indicate the regions within which the difference between our model and the experimental data is smaller than the uncertainty of the experimental results,*i*.*e*., $|TD_r-TY_r| \le 0.06$, as shown in Fig. 3. For a fixed external magnetic field $B=50 \mu T$ and $a_1=523.3 \mu$T, our model can predict the experimental results with quite broad ranges for the HFCC between the unpaired electron and Li nuclear spin, $a_2$, the RP reaction rate, *k*, the RP spin relaxation rate, *r*, namely $|a_2| \in [210, 500] \mu$T , $k \in [1.\times 10^8, 1\times 10^9]$ s$^{-1}$, and $r \in [1\times 10^6, 6\times 10^7]$ s$^{-1}$, see Fig. 3. The predicted values for the lithium HFCC overlaps with the range that we find for Li${^{+}}$-O2${^{.-}}$ from our DFT calculations.

## Discussion

Our principal goal in this project was to probe whether the RPM can be the underlying mechanism behind the isotopic dependence of lithium’s effectiveness for the hyperactivity treatment observed by Ettenberg et al.^11^. Our results support such a mechanism. A simple radical model with a set of reasonable parameters can reproduce the experimental findings.

We proposed that the [FADH${^{.}}$... O2${^{.-}}$] complex is the naturally occurring RP in the circadian center of the brain and lithium interacts with the radical electron on superoxide. This is motivated by the observations^51^ that one of the possible pathways for Li’s effects is Li’s direct influence on the suprachiasmatic nucleus–a region in the brain containing cryptochrome protein–, that bipolar disorder is associated with imbalances in the ROS level^14^, and that the lithium treatment modulates the oxidative stress level^7,15,20,21^. By varying the RP spin-coherence relaxation rate, RP reaction rate, and hyperfine coupling parameters our model reproduced the experimental findings of Ettenberg et al.^11^. The predicted range for the lithium hyperfine coupling constant in Li${^{+}}$-O2${^{.-}}$ overlaps with our DFT calculations.

Let us note that the RPM model could be adapted for other pathways by which lithium could affect BD. For example, it has also been proposed that Li could exert its effects on BD via increasing glutamate re-uptake at the NMDA receptor^68^. Inspired by the RPM’s potential role in xenon-induced anesthesia^35^, where there the NMDA receptor has been proposed as the target site of xenon, a similar scenario could be envisioned here. For example, oxygen might oxidize Trp in the NMDA receptor and result in the formation of [TrpH${^{.+}}$...O2${^{.-}}$] RPs, and lithium might modulate the S-T interconversion of the RPs by coupling to the unpaired electron of O2${^{.-}}$.

There remains a question on the feasibility for the O2${^{.-}}$ radical to be involved in the RPM in this scenario due to its expected fast spin relaxation rate *r*. In the context of magnetoreception, the superoxide-based RP model has been discussed by Hogben et al.^44^ and Player and Hore^69^. The authors argue that due to fast molecular rotation free O2${^{.-}}$ has a spin relaxation lifetime on the orders of 1 ns. The relaxation rate requirement calculated by our model yields *r* about two orders of magnitude slower than this value, see Fig. 3. However, the same authors have also noted that this fast spin relaxation of free superoxide can be can be lowered if the molecular symmetry is reduced and the angular momentum quenched by its biological environment. Moreover, Kattnig^70,71^ proposed that such fast spin relaxation of O2${^{.-}}$ could be, in effect, reduced by the involvement of scavenger species around O2${^{.-}}$. For example, it has been suggested that, in the RPM, Trp${^{.}}$ could act as a scavenger molecule for O2${^{.-}}$^42^. It should also be noted that it is often assumed that in the RP formation process oxygen starts out in its triplet ground state and consequently the initial state of the RPs in [FADH${^{.}}$... O2${^{.-}}$] would be a triplet state, as opposed to the singlet state considered here. However, there is a possibility that the initial state of O2 is the excited singlet state^72–74^ (which is a biologically relevant ROS) instead. Moreover, spin-orbit coupling^75,76^ could also transform the initial states from triplet to singlet states. Alternatively, other RP constituents could be considered instead of O2${^{.-}}$ to explain isotopic effects within the framework of the RPM.

The predicted dependence of the triple yield on changes of external magnetic field in Fig. 4 indicates that the effectiveness of the Li treatment could be enhanced by applying external magnetic fields. It would be of interest to conduct such experiments *in vivo* to explore the impact of the external magnetic field on the effectiveness of the different isotopes of lithium for hyperactivity treatment. It would also be of interest, to explore isotopic nuclear-spin effects of key elements of the biological environment (particularly oxygen, but also e.g. nitrogen, carbon, and hydrogen) in experiments on the lithium effectiveness on hyperactivity.

In summary, our results suggest that quantum entanglement might lie behind the mechanism of lithium treatment for BD, similarly to magnetoreception in animals^33^ and (as recently proposed) xenon-induced anesthesia^35^.

This also raises the question whether the RPM could play a role in other mental disorders, and could lead to new approaches to treatment and improving efficiency of medications^77^, specifically for illnesses that have been shown to be associated with oxidative stress, such as Alzheimer’s^78,79^, Schizophrenia^80–82^, and Parkinson’s^83^. Further, it is known that light exposure affects mood and emotions^84–89^, and light is required for the formation of RPs in Crys^39,69^. It therefore seems that RPM could also elucidate the effects of light exposure on mood. Similarly, RPM may provide explanations for the anti-depressant effect of vitamin D^90–92^ and its effects on the modulation of ROS production^93,94^. Given that the RPM is typically associated with isotope and magnetic field effects, it would be of great interest to search for such effects for other neurological medications.

Memory, learning, and subjective experience are affected by moods and emotions, and it has been proposed that cognition and subjective experience may be related to large-scale entanglement^60,95–98^. In this highly speculative context, entangled RPs could play crucial roles as sources of entanglement, and the present work could be viewed as another piece of evidence consistent with this idea in addition to Ref.^35^. In particular, superoxide radicals can give rise to singlet oxygen, which can emit photons^99^. These photons could serve as quantum messengers to establish long-distance connections^100^ that might be essential for consciousness^101^.

## Methods

### DFT analysis

The ORCA package^102^ was used for our Li${^{+}}$-O2${^{.-}}$ DFT calculations, and the molecular structure was optimized using the dispersion-corrected PBE0 functional and def2-TZVP basis set.

**Table 3:** HFCC $|a_2|$($\mu$T) using different DFT fuctionals.

Functional	$|a_2|$
BHLYP	282
RI-B2GP-PLYP	224.4
B3LYP	210.8
B3LYP/G	209.8
PBE0	167.2
B3PW91	157.7
PW6B95	208.4

The orbitals obtained from the optimization calculations were used to calculate orbital energies as well as the hyperfine coupling constant $a_2$. Using various DFT functionals, we obtained $|a_2| \in [157.7, 282] \mu$T, see Table 3. The calculation with the double hybrid functional^103^ RI-B2GP-PLYP with def2-QZVPP basis-set resulted $|a_2| = 224.4 \mu$T, which is in the range that our RPM model predicts. We used this value in our computations. Relativistic effects were treated by a scalar relativistic Hamiltonian using the zeroth-order regular approximation (ZORA)^104^. Solvent effects were considered by using the conductor-like polarizable continuum model (CPCM)^105^, with a dielectric constant of 2.

## Data Availability

The generated datasets and computational analysis are available from the corresponding author on reasonable request.
